# Expression profiling of N6-methyladenosine-modified mRNA in PC12 cells in response to unconjugated bilirubin

**DOI:** 10.1007/s11033-023-08576-1

**Published:** 2023-06-28

**Authors:** Jinfu Zhou, Sining Liao, Chenran Zhang, Jinying Luo, Guilin Li, Huangyuan Li

**Affiliations:** 1 Department of Preventive Medicine, School of Public Health, Fujian Medical University, Fuzhou, 350122 Fujian Province China; 2 Medical Genetic Diagnosis and Therapy Center, Fujian Maternity and Child Health Hospital College of Clinical Medicine for Obstetrics & Gynecology and Pediatrics, Fujian Medical University, Fuzhou, 350001 Fujian Province China; 3 Fujian Provincial Key Laboratory of Environmental Factors and Cancer, School of Public Health, Fujian Medical University, Fuzhou, 350122 Fujian Province China; 4 Obstetrics and Gynecology Department, Fujian Maternity and Child Health Hospital College of Clinical Medicine for Obstetrics & Gynecology and Pediatrics, Fujian Medical University, Fuzhou, 350001 Fujian Province China; 5 Key Laboratory of Environment and Health, School of Public Health, Fujian Medical University, Fuzhou, 350122 Fujian Province China

**Keywords:** *N*^6^-methyladenosine, Methylated RNA immunoprecipitation sequencing, Methylation, mRNA profile, Neurotoxicity, Unconjugated bilirubin

## Abstract

**Background:**

Abnormal methylation of *N*^6^-methyladenosine (m^6^A) is reportedly associated with central nervous system disorders. However, the role of m^6^A mRNA methylation in unconjugated bilirubin (UCB) neurotoxicity requires further research.

**Methods:**

Rat pheochromocytoma PC12 cells treated with UCB were used as in vitro models. After the PC12 cells were treated with UCB (0, 12, 18, and 24 µM) for 24 h, the total RNA m^6^A levels were measured using an m^6^A RNA methylation quantification kit. The expression of m6A demethylases and methyltransferases was detected through western blotting. We determined the m^6^A mRNA methylation profile in PC12 cells exposed to UCB (0 and 18 µM) for 24 h using methylated RNA immunoprecipitation sequencing (MeRIP-seq).

**Results:**

Compared with the control group, UCB (18 and 24 µM) treatment decreased the expression of the m^6^A demethylase ALKBH5 and increased the expression of the methyltransferases METTL3 and METTL14, which resulted in an increase in the total m^6^A levels in PC12 cells. Furthermore, 1533 m^6^A peaks were significantly elevated and 1331 peaks were reduced in the UCB (18 µM)-treated groups compared with those in the control group. Genes with differential m^6^A peaks were mainly enriched in protein processing in the endoplasmic reticulum, ubiquitin-mediated proteolysis, cell cycle, and endocytosis. Through combined analysis of the MeRIP-seq and RNA sequencing data, 129 genes with differentially methylated m^6^A peaks and differentially expressed mRNA levels were identified.

**Conclusion:**

Our study suggests that the modulation of m^6^A methylation modifications plays a significant role in UCB neurotoxicity.

**Supplementary Information:**

The online version contains supplementary material available at 10.1007/s11033-023-08576-1.

## Introduction

Hyperbilirubinemia is commonly present in newborn babies. Approximately one in ten neonates develop hyperbilirubinemia in the first 2 weeks of life [1]. Abnormal accumulation of unconjugated bilirubin (UCB) in the blood of newborns can lead to bilirubin crossing the blood–brain barrier and causing brain damage. UCB neurotoxicity can cause irreversible damage to specific regions of the brain, including the globus pallidus, hippocampus, and subthalamic nucleus, and its sequelae mainly include learning disabilities, movement disorders, mental retardation, and cerebral palsy [2]. At present, the potential mechanisms of bilirubin-induced nerve cell damage mainly include oxidative stress [3], apoptosis [4], endoplasmic reticulum (ER) stress [5–7], autophagy [8], and neuroinflammation [9]. However, the fundamentals of bilirubin neurotoxicity are not completely understood and require further elucidation.

Epigenetic modifications play a significant role in UCB neurotoxicity. DNA methylation and histone modifications are also involved in UCB neurotoxicity [10, 11]. DNA methylation may play a critical role in bilirubin-induced neuronal injury. In vitro exposure to high UCB concentrations led to changes in methylation levels of genes in neuronal cells of neonatal Sprague–Dawley rats, and these levels were negatively correlated with gene expression levels [10]. H3K14 acetylation is reportedly involved in bilirubin-induced cerebellar hypoplasia [11]. Currently, the study of epigenetics in bilirubin-induced nerve cell injury is limited to DNA-level epigenomics, whereas the correlation between RNA epigenomics and bilirubin-induced nerve cell injury has not been reported.

*N*^6^-methyladenosine (m^6^A) is one of the most abundant and prevalent chemical modifications, especially in eukaryotic mRNA and long noncoding RNAs [12] m^6^A is mainly localized in the 3′-untranslated region (UTR) and coding sequence (CDS) of mRNA, and its common sequence is RRACH (R = G or A; H = A, C, or U) [13]. m^6^A regulates the posttranscriptional processing of mRNAs by affecting splicing, nucleation, degradation, and translation. This modification is regulated by methyltransferases (“writers”) and demethylases (“erasers”). “Writers” are complexes composed of various components, including the RNA methylase m^6^A methyltransferase 3 (METTL3), METTL14, WTAP, and RBM15, among which the most critical molecule with catalytic active domains is METTL3 [14, 15]. “Erasers” include FTO and the RNA demethylase alkB homolog 5 (ALKBH5), which demethylate m^6^A residues. Therefore, the combined action of “writers” and “erasers” makes m^6^A modification a dynamic and reversible process.

In the mammalian brain, m^6^A modification is essential for cortical neurogenesis [16], cerebellar development [17], and adult neural stem cell proliferation and differentiation [15]. However, abnormal m^6^A methylation is associated with the pathogenesis of central nervous system (CNS) diseases. For instance, changes in m^6^A modification are not only involved in chronic CNS diseases, such as Alzheimer’s disease [18], Parkinson’s disease [19], and depression [20], but also in acute brain injuries, such as traumatic brain injury [21] and stroke [22]. However, whether m^6^A RNA methylation plays a role in bilirubin neurotoxicity remains unknown.

To investigate the effect of m6A RNA methylation in UCB neurotoxicity, we evaluated the changes in the m^6^A mRNA methylation profile in PC12 cells exposed to UCB using methylated RNA immunoprecipitation (IP) sequencing (MeRIP-seq). We further analyzed the combined MeRIP-seq and RNA sequencing (RNA-seq) data. Herein, we report, for the first time, UCB-induced changes in the transcriptome m^6^A profile in neural cells and provide a fundamental basis for further studies.

## Methods

### Cell culture and treatment

PC12 cells (Wuhan Boster Biological Technology, Ltd., Wuhan, China) were isolated from the pheochromocytoma of the rat adrenal medulla. Cells were cultured in Dulbecco’s modified Eagle medium (Gibco, Carlsbad, CA, USA) supplemented with fetal bovine serum (Gibco, Carlsbad, CA, USA; 10%) and penicillin–streptomycin (1%). For the 3-(4,5-dimethylthiazol-2-yl)-2,5-diphenyltetrazolium bromide cell viability assay (F5655; Sigma-Aldrich, St. Louis, MO, USA; Fig. S1), cells were exposed to various concentrations of bilirubin (0, 12, 18, and 24 µM) for 24 h, and the total m^6^A levels and protein expression of RNA demethylases and methylases were determined. For MeRIP- and RNA-seq, cells were exposed to 0 and 18 µM bilirubin in triplicate to analyze m^6^A epitranscriptomic and RNA transcriptomic alterations.

### Analysis of total m^6^A levels

Total RNA m^6^A levels of the bilirubin and control groups were measured using an m^6^A RNA methylation quantification kit (P-9005; EpiGentek, Farmingdale, NY, USA). Briefly, the RNA-binding reagent was mixed with the total RNA extracted from the cells. Subsequently, the capture and detection antibodies were used to measure the m^6^A signal, which was enhanced using the enhancement solution. The final absorbance was measured at 450 nm to calculate the amount of m^6^A as per the manufacturer’s instructions.

### MeRIP-seq and RNA-seq

RNA- and MeRIP-seq experiments were performed by Guangzhou Epigenetic Biotechnology Co., Ltd. (Guangzhou, China). Briefly, total RNA was extracted using the TRIzol™ reagent (Invitrogen, Waltham, MA, USA) for subsequent experiments. Thereafter, mRNA was isolated from the total RNA samples and digested into 100–200-nucleotide fragments. Methylated RNA IP was performed using the Epi™ m^6^A IP kit (R1804; Epibiotek, Guangzhou, China). The partial nucleotide fragments were used as an input, and both the input and m^6^A IP samples were fragmented to a size of 150 bp. Illumina NovaSeq 6000 sequencer (Illumina, San Diego, CA, USA) was used to perform the sequencing.

The quality of the mRNA sequencing data was verified using FastQC (v0.11.7), and differential m^6^A peaks (*P* < 0.05) between the bilirubin and control groups were analyzed using the exomePeak R package (v2.13.2). Motifs of the m^6^A peak sequences were determined using HOMER. Gene Ontology (GO) terms and Kyoto Encyclopedia of Genes and Genomes (KEGG) pathways were analyzed using the respective databases.

### Western blotting

A radioimmunoprecipitation assay lysis buffer (Beyotime, Beijing, China) was supplemented with phenylmethylsulfonyl fluoride (Phygene, Fuzhou, China) and 1% protease inhibitor (MedChemExpress, NJ, USA) to extract the proteins from the PC12 cells. Then, the protein supernatant was mixed with 5× loading buffer and denatured at 95 °C. Proteins of different molecular weights were separated using sodium dodecyl sulfate-polyacrylamide gel electrophoresis and transferred to 0.2 μm polyvinylidene fluoride (PVDF) membranes. Next, proteins were blocked with 5% non-fat milk for 60 min. PVDF membranes with protein layers were incubated overnight at 4 °C with primary antibodies against METTL3 (A8307; ABclonal, Wuhan, China) (1:1 000), METTL14 (A8530; ABclonal) (1:1 000), ALKBH5 (ab195377; Abcam, Cambridge, MA, USA) (1:2 000), and FTO (A1438; ABclonal) (1:1 000). The PVDF membranes were then incubated with secondary antibodies (AS014; ABclonal) at 37 °C for 60 min. Image J software (v. 1.53) was used to quantify the grayscale of the protein bands.

### Real-time quantitative PCR (RT-qPCR)

The sequences of primers to amplify the regions of the ALKBH5, FTO, METTL3, and METTL14 genes, with the GAPDH gene was as an internal control gene, were designed and synthesized by Sangon Bioengineering (Shanghai) Co., LTD and are shown in Table S1. PC12 cells were treated with bilirubin (0, 12, 18, and 24 µM) for 24 h. After that, RNA was extracted, cDNA was synthesized, and RT-qPCR reaction was conducted using the SYBR Green master mix (TaKaRa Biotech, Tokyo, Japan) based on the instructions from the supplier. Data were analyzed using the 2^−ΔCt^ method.

### Statistical analysis

Data were analyzed using the GraphPad Prism V8.0 software (GraphPad Software, San Diego, CA, USA). Data from the western blot analyses are presented as the mean ± standard error of the mean. At least three separate experiments were performed for each study group, and the results were analyzed using one-way analysis of variance. *P* < 0.05 indicates that the difference is significant.

## Results

### UCB induced the alteration of total m^6^A levels in PC12 cells resulting from the changed expression of the m^6^A methyltransferase and demethylase

**Fig. 1:**
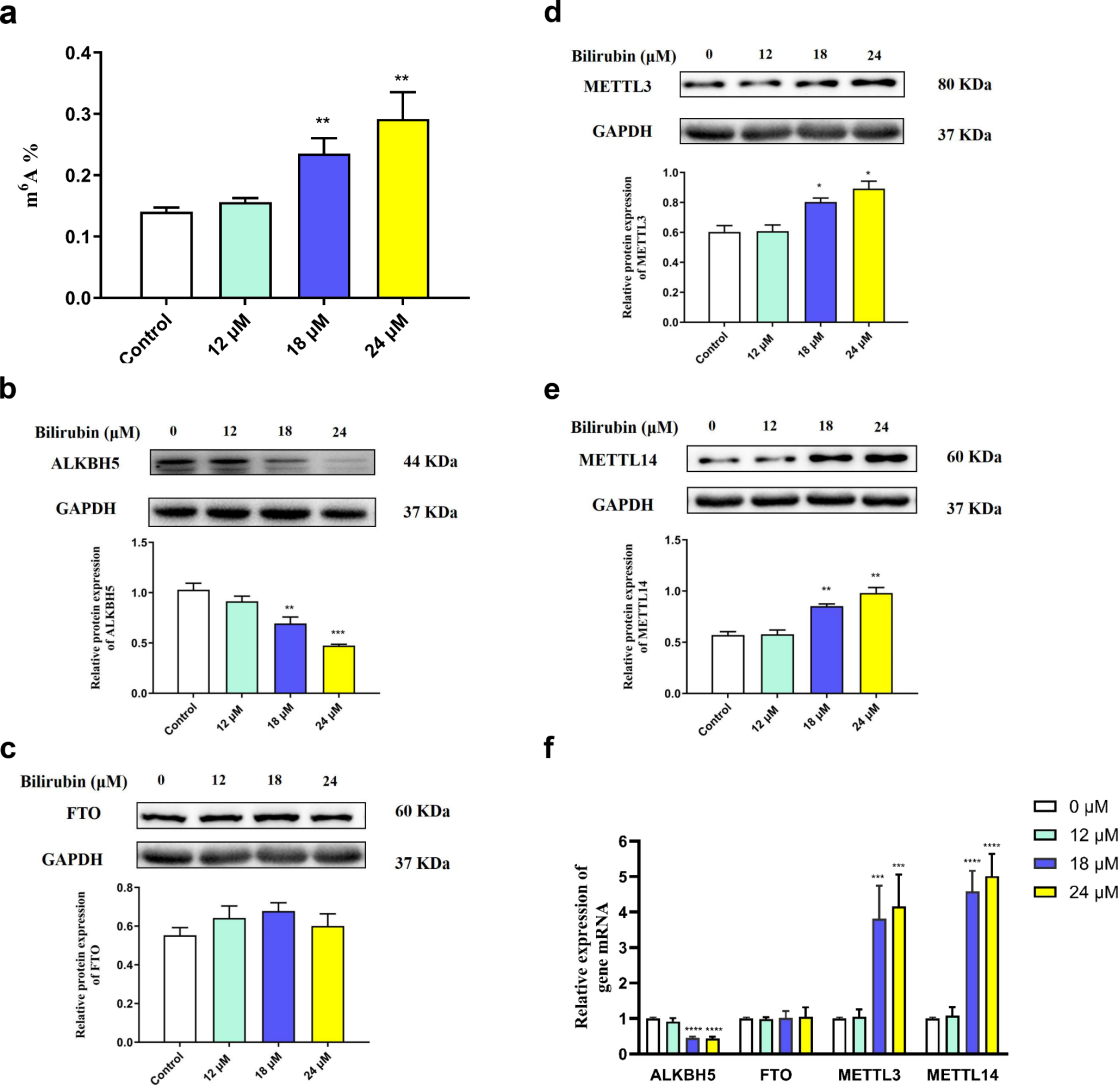
Effect of m^6^A methylation modification in PC12 cells exposed to bilirubin. (**a**) Total m^6^A level. (**b, c**) The protein expression of the RNA demethylases ALKBH5 and FTO. (**d, e**) The protein expression of the RNA methylases METTL3 and METTL14. (**f**) The relative expression of ALKBH5, FTO, METTL14, and METTL3 mRNA. Data are presented as the mean ± SD, n = 3. *P < 0.05, **P < 0.01, ***P < 0.001, and ****P < 0.000 compared to the control group

Changes in the total m^6^A levels were measured in PC12 cells exposed to 12, 18, and 24 µM bilirubin for 24 h. We found that the total m^6^A levels in the experimental groups treated with 18 and 24 µM bilirubin were significantly higher than those in the control group (*P* < 0.05) (Fig. 1a). To elucidate the cause of hypermethylation in the bilirubin-treated groups, the levels of m^6^A methylases and demethylases were determined by performing western blotting. The results showed that the expression of the m^6^A modified demethylase ALKBH5 (Fig. 1b) significantly decreased and that of the m^6^A modified methyltransferases METTL3 (Fig. 1c) and METTL14 (Fig. 1d) increased after treatment with 18 or 24 µM bilirubin. However, the expression of the m^6^A demethylase FTO (Fig. 1e) remained unchanged. These results suggest that bilirubin exposure can promote m^6^A mRNA hypermethylation in PC12 cells. In addition, compared to that in the control group, the expression of the ALKBH5 mRNA significantly decreased in the PC12 cells when treated with 18 or 24 µM bilirubin, while the expression of the METTL3 and METTL14 mRNA increased (Fig. 1f).

### Differential m^6^A modification profiles in PC12 cells

**Fig. 2:**
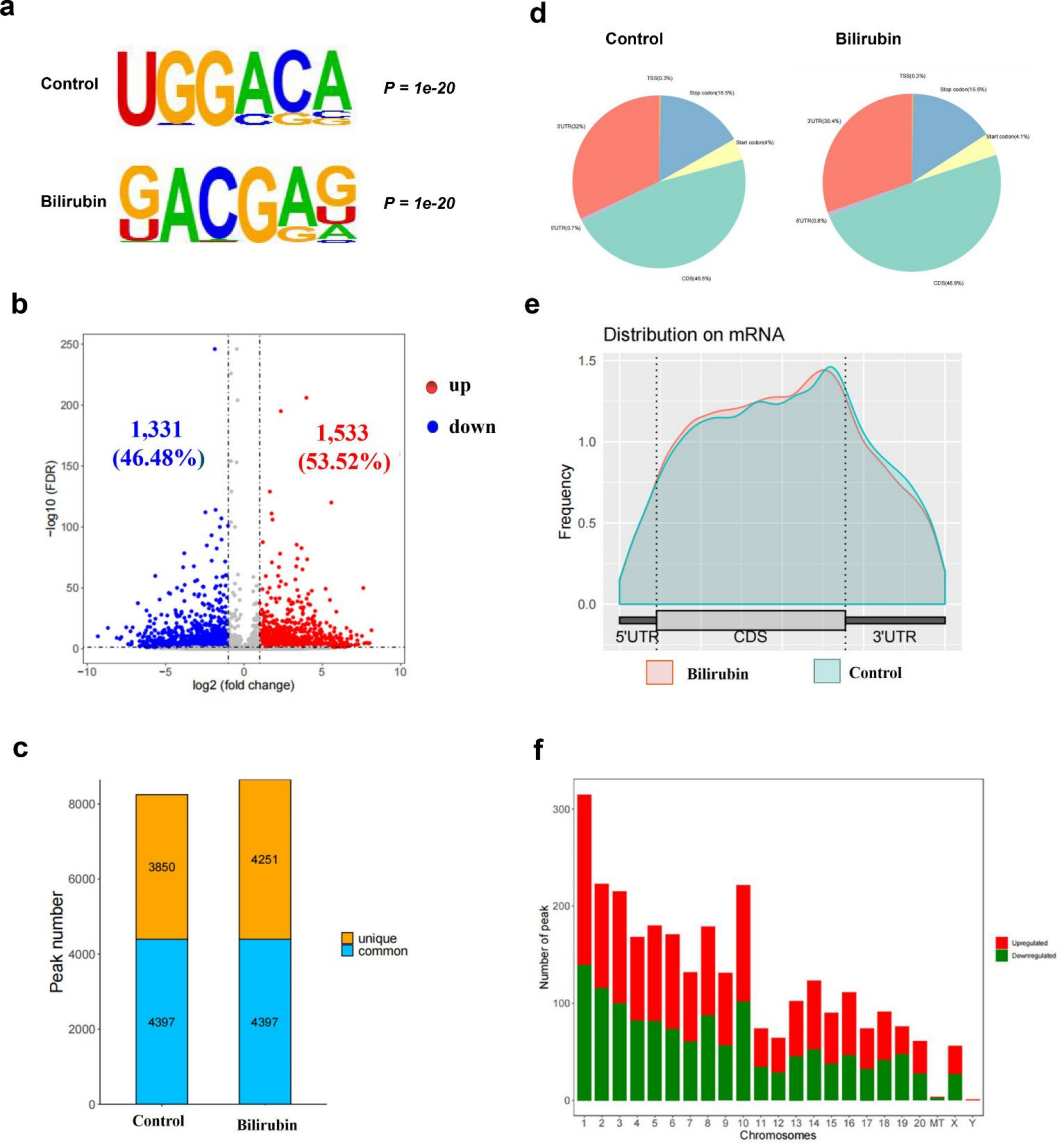
Differential m^6^A modification profiles in PC12 cells. (**a**) The sequence motif of m^6^A-containing peak regions in the bilirubin and control groups. (**b**) Volcano plots of differentially methylated mRNAs in the bilirubin group compared with those in the control group. (**c**) Unique and common peak numbers in the bilirubin and control groups. (**d**) The proportion of m^6^A peaks located at the mRNA transcripts. (**e**) The densities of m^6^A peaks in the bilirubin and control groups were compared to those in the 5′-UTR, CDS, and 3′-UTR regions. (**f**) Changes in the m^6^A peak quantity in different chromosomes after bilirubin treatment

Based on the results of the total m^6^A levels and protein expression of RNA demethylases and methylases, PC12 cells were exposed to 0 and 18 µM bilirubin in triplicate to analyze m^6^A epitranscriptomic alterations through MeRIP- and RNA-seq.

m^6^A modification was characterized using motif analysis. The consensus motif of the m^6^A peaks was identified to be G[G]AC[GA] (Fig. 2a), which was consistent with the specific motif of m^6^A methylation sites. The results suggested that 1533 m^6^A peaks were significantly elevated and 1331 peaks were reduced in the bilirubin-treated groups compared with those in the control group (fold change ≥ 1 and *P* < 0.05; Fig. 2b). The m^6^A methylation level of 10 methylated genes and 10 demethylated genes, listed in Table 1, was significantly altered under treatment with bilirubin in accordance with *diff.log2.fc*. In the control group, 8247 m^6^A peaks were recognized, among which 3850 were unique and 4397 were shared with the bilirubin group. Approximately 8600 m^6^A peaks were identified in the bilirubin group, among which 4251 were unique (Fig. 2c).

**Table 1:** Top 20 genes with altered m^6^A peaks

Gene name	chromosome	Peak star	Peak End	lg.p	diff.log2.fc	Regulation
Jak1	5	119,984,644	119,985,175	-6.01	-9.32	down
Smad4	18	69,628,141	69,634,083	-10.1	-8.68	down
Gpsm2	2	211,513,403	211,513,582	-2.96	-8.12	down
Mobp	8	128,841,916	128,842,306	-9.3	-7.91	down
Sbno1	12	37,627,019	37,628,226	-2.79	-7.82	down
Matr3	18	28,371,575	28,371,755	-12.1	-7.51	down
Nup155	2	57,250,191	57,251,998	-6.67	-7.39	down
Plcg1	3	156,747,621	156,748,564	-17.8	-7.24	down
Usp39	4	100,187,353	100,190,182	-8.38	-7.23	down
Pygb	3	146,611,545	146,612,871	-6.55	7.17	up
Slc38a2	7	138,089,664	138,089,875	-24.1	7.19	up
Ddx1	6	38,423,066	38,423,906	-13.6	7.23	up
Bcar1	19	43,935,829	43,936,009	-4.68	7.29	up
Pdgfrb	18	56,381,091	56,381,212	-2.93	7.52	up
Myo9b	16	19,745,273	19,746,952	-14.8	7.61	up
Nhlrc2	1	277,409,493	277,409,703	-4.94	7.67	up
Cdk12	10	86,157,936	86,158,116	-8.62	7.81	up
Rspry1	19	10,812,736	10,812,944	-9.3	7.91	up
Zhx3	3	156,776,897	156,777,106	-13	8.01	up
Dhrs9	3	55,647,252	55,647,491	-3.97	8.13	up

In general, m^6^A peaks in the control and bilirubin groups were distributed in the start codon, CDS, stop codon, and 3′-UTR and were mainly located in the CDS near the 3′-UTR. Compared with that in the control group, in the bilirubin group, the distribution of m^6^A peaks was increased in the CDS of mRNAs and decreased in the 3′-UTRs (Fig. 2d and e). Bilirubin also increased the number of m^6^A modification peaks in the CDS region.

### Functional analysis of differentially m^6^A methylated mRNAs in PC12 cells

**Fig. 3:**
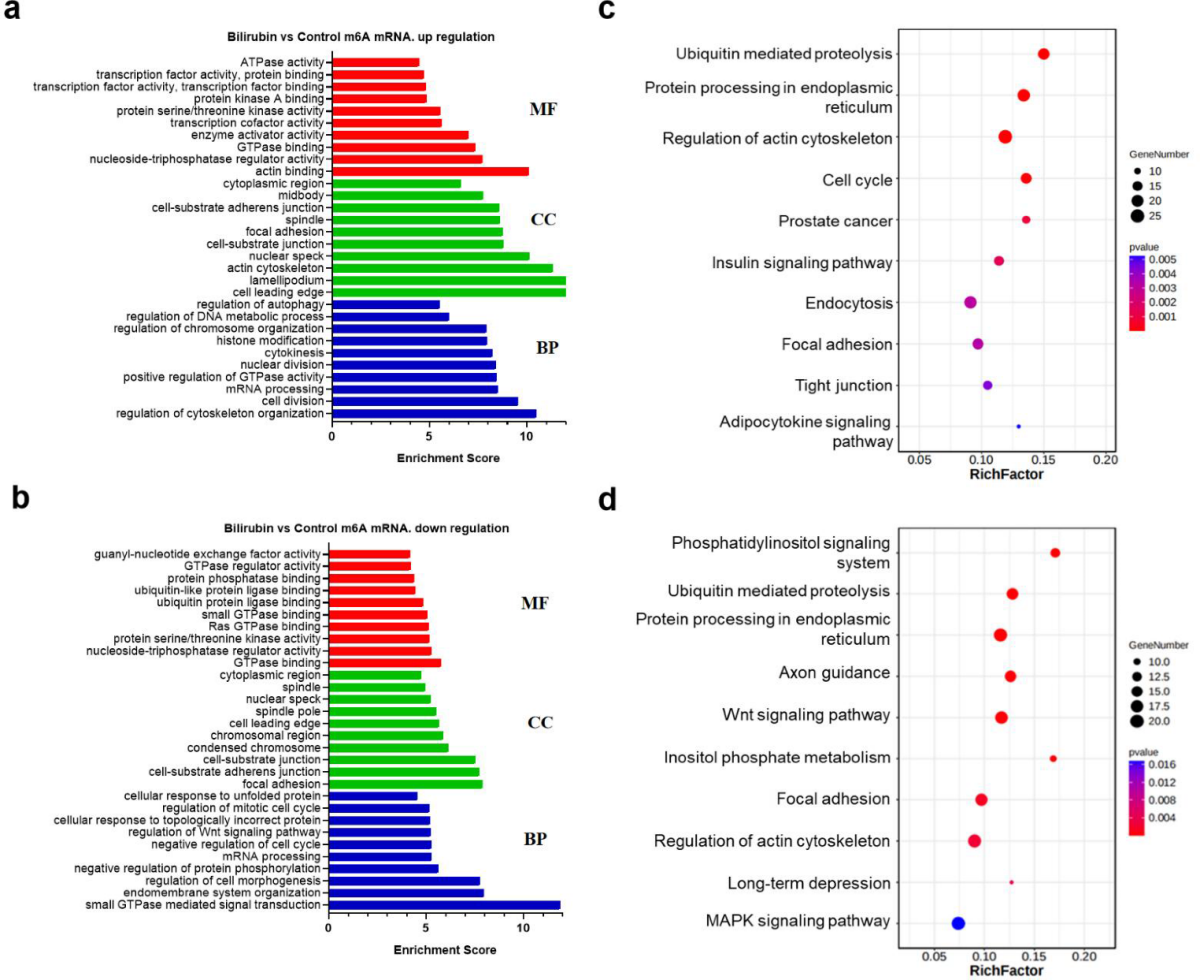
Functional analysis of differentially m^6^A-modified mRNAs in PC12 cells between the bilirubin and control groups. (**a**) GO terms of genes with upregulated m^6^A peaks. (**b**) GO terms of genes with downregulated m^6^A peaks. (**c**) KEGG pathway analyses of genes with upregulated m^6^A peaks. (**d**) KEGG pathway analyses of genes with downregulated m^6^A peaks

To determine which molecular signaling pathways and functions associated with neurotoxicity were affected by bilirubin, the differentially methylated transcripts were analyzed using GO functional and KEGG pathway analyses. GO enrichment analysis revealed that the genes with differentially m^6^A methylated peaks were involved in cytokinesis, cell division, mRNA processing, endomembrane system organization, transcription, protein kinase enzymes, and GTPase binding. Further analysis revealed the top 10 significantly enriched biological processes (BPs), cellular components (CCs), and molecular functions (MFs) of the genes with increased m^6^A peaks, which are shown in Fig. 3a, while GO analysis of genes with decreased m^6^A peaks is shown in Fig. 3b.

For the KEGG pathway analysis, we identified 28 and 30 pathways that involved the genes with upregulated and downregulated m^6^A methylated peaks, respectively. The genes with elevated m^6^A peaks in the bilirubin group were significantly correlated with ubiquitin-mediated proteolysis, protein processing in the ER, cell cycle, and endocytosis (Fig. 3c). The genes with reduced peaks were significantly related to the phosphatidylinositol signaling system, protein processing in the ER, and the Wnt and mitogen‑activated protein kinase signaling pathways (Fig. 3d).

### Cluster analysis of differentially expressed mRNAs in PC12 cells

**Fig. 4:**
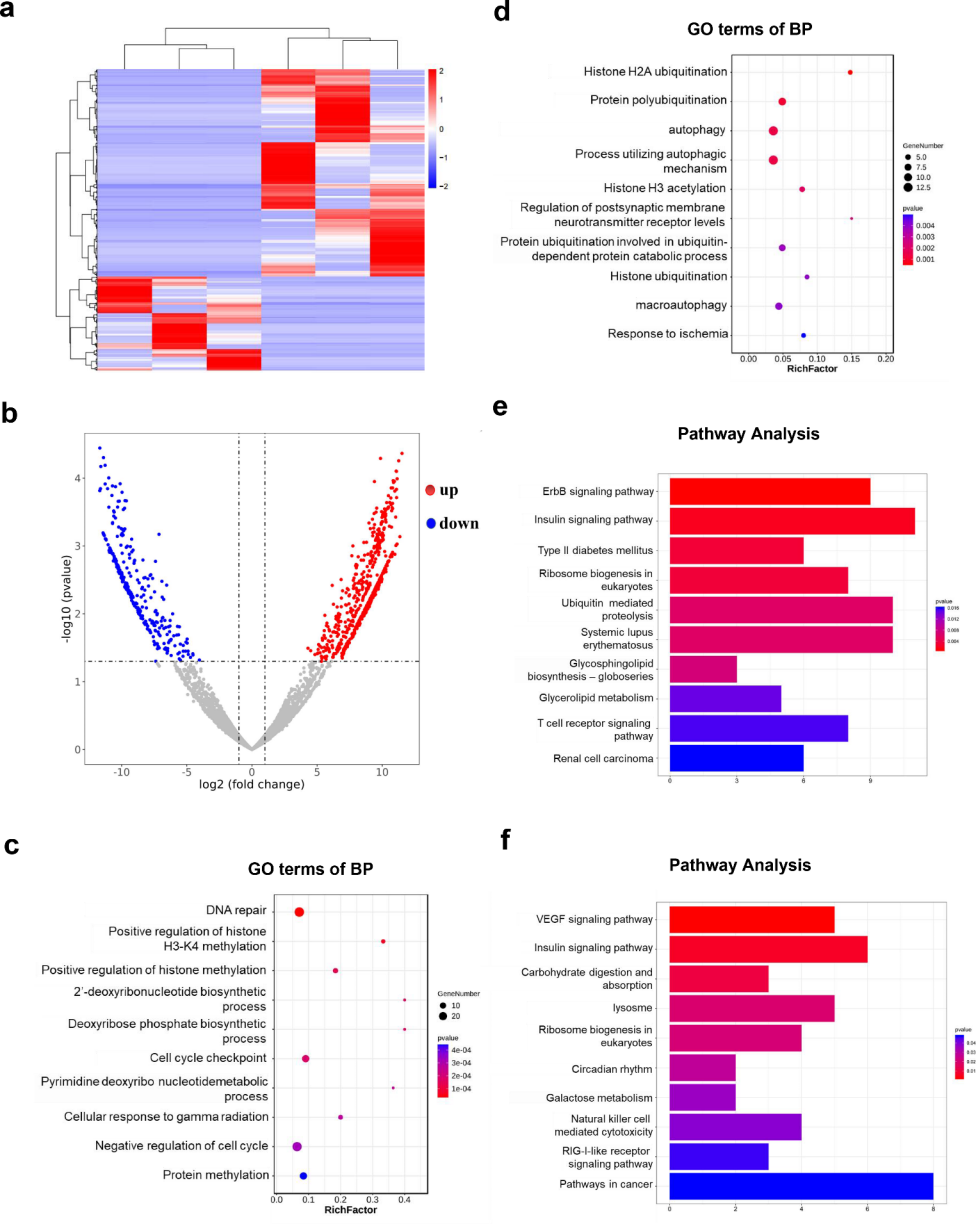
Cluster analysis of differentially expressed mRNAs in PC12 cells between the bilirubin and control groups. (**a**) Heatmap of differentially expressed mRNAs in the bilirubin group compared with the control group. (**b**) Scatter plots showing the mRNA data (fold changes ≥ 2 and *P* < 0.05). (**c**) GO analysis of the upregulated genes in the bilirubin group. (**d**) GO analysis of the downregulated genes in the bilirubin group. (**e**) KEGG pathway analyses of the upregulated genes in the bilirubin group. (**f**) KEGG pathway analysis of the downregulated genes in the bilirubin group

RNA-seq was used to analyze mRNA transcriptional profiles in PC12 cells after bilirubin treatment. Significant differences could be observed in the heat map of mRNA expression patterns between the bilirubin and control groups (Fig. 4a). A volcano map revealed that the expression profiles of 940 mRNAs, including 647 upregulated and 293 downregulated mRNAs, were significantly dysregulated in the bilirubin group compared with those in the control group (fold change > 2 and *P* < 0.05; Fig. 4b). Subsequently, in order to evaluate what functions these differentially expressed mRNAs play after bilirubin treatment, we performed GO enrichment and KEGG pathway analyses. The results indicated that the significant differentially expressed mRNAs between the bilirubin and control groups (fold change > 2 and *P* < 0.05) were mainly associated with autophagy, histone H3 acetylation, and DNA repair (Fig. 4c and d). In addition, VEGF signaling pathway, ribosome biogenesis, and lysosomes were found to play a vital role in the bilirubin-treated group (Fig. 4e and f).

### Combined analysis of GO and KEGG pathways with MeRIP- and RNA-seq data

**Fig. 5:**
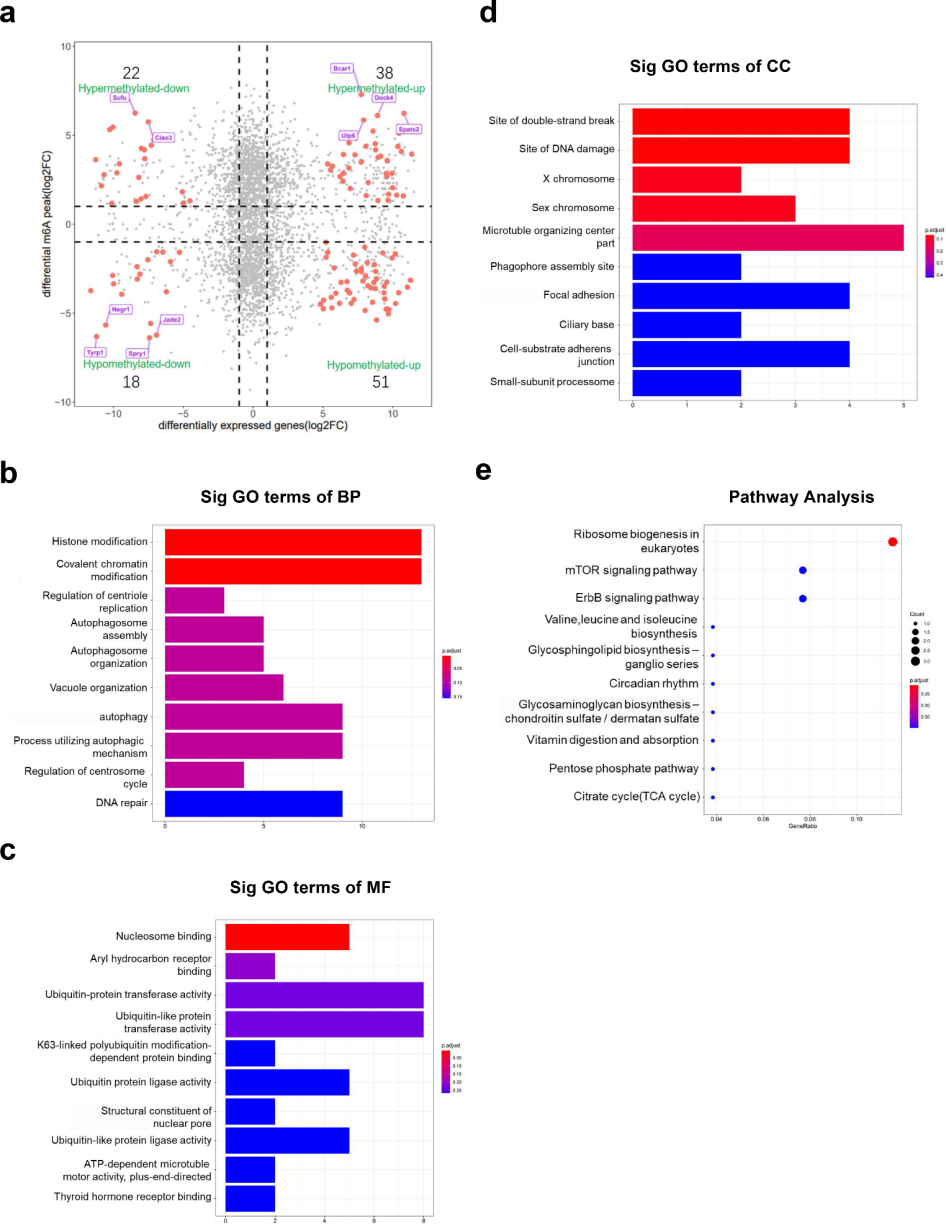
Combined functional analysis of differentially expressed genes between MeRIP-seq and RNA-seq data. (**a**) Four-quadrant plot showing the genes with differential changes in both m^6^A modification level and post-transcriptional mRNA level in the bilirubin group compared with the control group. The red dots represent genes with significant differences in m^6^A modification and mRNA expression between the bilirubin and control groups. (**b**) GO analysis of BP performed for differentially expressed genes identified through both MeRIP- and RNA-seq analyses. (**c**) GO analysis of MF of significantly differentially expressed genes identified through both MeRIP- and RNA-seq analyses. (**d**) GO analysis of CC of significantly differentially expressed genes identified through both MeRIP- and RNA-seq analyses. (**e**) KEGG pathway analysis of the MeRIP-seq and RNA-seq data

Through a combined analysis of the MeRIP- and RNA-seq results, 129 genes with differentially methylated m^6^A peaks and differential mRNA levels were divided into four parts, as shown in Fig. 5a. This study showed that 38, 51, 22, and 18 genes were upregulated and hypermethylated, upregulated and hypomethylated, downregulated and hypermethylated, and downregulated and hypomethylated, respectively. GO analysis also revealed that these genes were mainly related to the histone modification pathway (GO term: BP; Fig. 5b), nucleosome adhesion (GO term: MF; Fig. 5c), and protein binding (GO term: CC; Fig. 5d). The KEGG pathway analysis showed that these genes were mainly enriched in ribosome biogenesis in eukaryotes and the mTOR signaling pathway (Fig. 5e).

### Network construction of major enriched pathway genes in bilirubin-treated PC12 cells

**Fig. 6:**
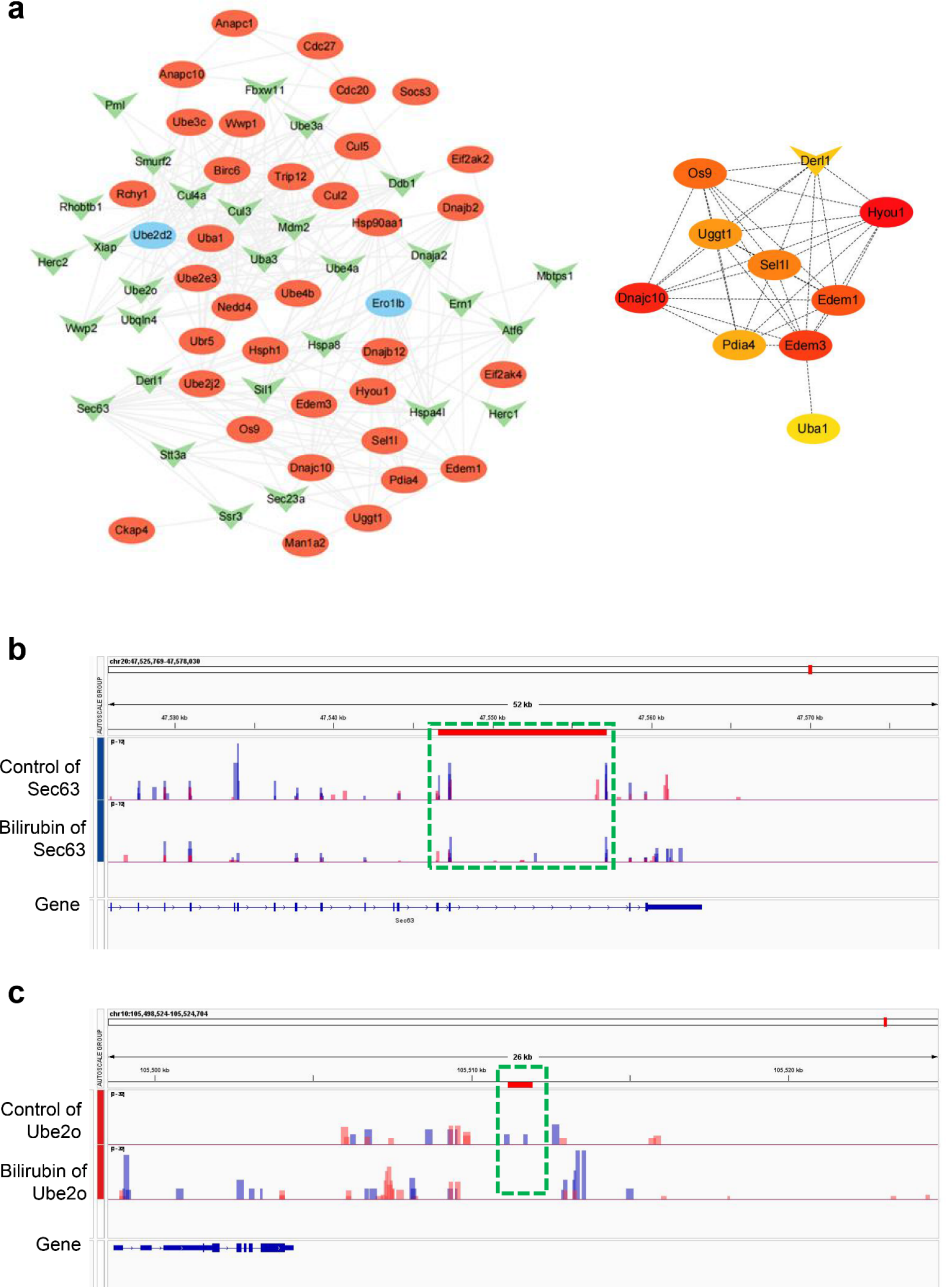
The differentially m^6^A-modified mRNA network in ubiquitin-mediated proteolysis and protein processing in endoplasmic reticulum pathways. (**a**) The interaction network of differentially m^6^A-modified mRNA in the ubiquitin-mediated proteolysis pathway and in protein processing of endoplasmic reticulum pathways. The genes shown in red are m^6^A hypermethylated genes and those shown in green are m^6^A hypomethylated genes. Genes showed in blue can interact with m^6^A differentially expressed genes, not m^6^A differentially expressed genes in MeRIP-seq data. The remaining genes represent the top 10 genes calculated by the number of nodes in the interaction network of differentially m^6^A-modified mRNA in the ubiquitin-mediated proteolysis pathway and in protein processing of the endoplasmic reticulum pathway. (**b, c**) *Sect. 63* and *Ube2o* transcripts in the UCB and control groups. Input and IP m^6^A are displayed as blue and red, respectively

Both upregulated and downregulated m^6^A-modified genes were enriched in ubiquitin-mediated proteolysis and protein processing in the ER as per KEGG pathway analysis. mRNA interaction networks were constructed using the Cytoscape software to investigate the effect of bilirubin-induced regulation of m^6^A modification regulation on ubiquitin-mediated proteolysis and protein processing in the ER (Fig. 6a). According to the degree of the differential expression of these m^6^A-modified genes in the protein processing in the ER and ubiquitin-mediated proteolysis pathways (Tables 2 and 3), *Sect. 63* and *Ube2o* were selected, respectively, and the m^6^A methylation sites and peak intensities were displayed using the IGV software (Fig. 6b and c).

**Table 2:** Top 10 altered m^6^A genes related to protein processing in the ER

Gene name	chromosome	Peak star	Peak End	lg.p	diff.log2.fc	Regulation
Section 63	20	47,560,798	47,561,068	-8.69	-6.67	down
Ero1b	17	90,710,794	90,711,154	-8.62	-4.85	down
Sec23a	6	80,061,005	80,062,850	-35.8	-4.56	down
Ssr3	2	156,012,961	156,013,140	-28.1	-4.34	down
Dnaja2	19	22,586,432	22,587,544	-5.97	-4.17	down
Ern1	10	94,652,644	94,681,854	-3.69	-4.08	down
Ube2d3	2	240,654,769	240,655,187	-5.7	3.96	up
Ube4b	5	166,214,129	166,215,524	-11	4.25	up
Dnajb2	9	82,437,551	82,439,401	-9.31	4.33	up
Dnajb12	20	29,455,755	29,457,342	-2.69	5.02	up

**Table 3:** Top 10 altered m^6^A genes related to ubiquitin-mediated proteolysis

Gene name	chromosome	Peak star	Peak End	lg.p	diff.log2.fc	Regulation
Ube2o	10	105,511,135	105,511,929	-6.55	-6.39	down
Herc1	8	72,116,404	72,117,022	-4.45	-5.83	down
Ube3a	1	116,648,732	116,656,481	-6.65	-5.81	down
Pml	8	63,016,204	63,018,743	-2.98	-4.58	down
Ube4a	8	49,264,827	49,269,334	-11.4	-4.1	down
Ubr5	7	76,808,850	76,810,827	-7.92	3.71	up
Ube2d3	2	240,654,769	240,655,187	-5.7	3.96	up
Ube4b	5	166,214,129	166,215,524	-11	4.25	up
Socs3	10	106,975,801	106,976,040	-17.7	4.68	up
Anapc10	19	31,815,766	31,815,916	-4.85	5.67	up

## Discussion

In the present study, we first investigated the expression of m^6^A methylation-modifying enzymes and revealed the m^6^A landscape of mRNAs in UCB-treated PC12 cells using high-throughput sequencing. PC12 cells are one of the most common models used in neuroscience studies exploring the latent mechanisms of action of neurotoxins [23], including those of bilirubin neurotoxicity [24]. Therefore, PC12 cells were selected as a model to study the potential neurotoxicity of bilirubin in the current study.

We observed that bilirubin treatment caused downregulation of the m^6^A demethylase ALKBH5 (Fig. 1b) and upregulation of the methyltransferases METTL3 and METTL14 (Fig. 1c and d). This resulted in increased m^6^A levels and led to an altered m^6^A expression profile in bilirubin-treated PC12 cells. Stable m^6^A methylation modifications are crucial in maintaining CNS function. In the mammalian nervous system, m^6^A methylation participates in various physiological functions, such as neuronal development, synaptic plasticity, learning and memory, addiction and reward learning, and stress responses [25]. Knockdown of *FTO* in the mouse prefrontal cortex and hippocampus led to enhanced m^6^A levels and the maintenance of fear memory and loss of learning ability [26, 27]. Specific differential expression of METTL13 and FTO and differential m^6^A methylation in different brain regions (medial prefrontal cortex and basolateral and central amygdala) were observed in mice during a stress response, which was consistent with the expression of glucocorticoids [28]. ALKBH5 overexpression inhibits cell proliferation via the Wnt/β-catenin signaling pathway, and METTL3 downregulation also reduces cell proliferation and increases apoptosis by inhibiting the Wnt/β-catenin signaling pathway [29]. *METTL14* knockdown significantly reduced proliferation and promoted the differentiation of mouse embryonic neural stem cells, thereby inhibiting their self-renewal ability [30]. These results indicate that m^6^A modification is involved in brain-related pathophysiology. Notably, we also found a specific mRNA m^6^A methylation profile in PC12 cells exposed to bilirubin, which suggests that mRNA m^6^A methylation modifications may be involved in bilirubin neurotoxicity.

We detected many differentially methylated mRNAs associated with several significant biological pathways. KEGG pathway analyses illustrated that ER protein processing was regulated by upregulated or downregulated genes with m6A-modified sites in bilirubin-treated PC12 cells. ER stress is closely related to bilirubin neurotoxicity [5–7]. For instance, a transcriptome analysis identified that more than 50 genes were directly involved in ER stress response in SHSY5Y cells with UCB treatment [5]. Bilirubin induced the expression of ER stress-related genes and activated the unfolded protein response, which ultimately caused neurological dysfunction syndrome [6]. UCB upregulated key proteins in ER stress-related pathways and induced mitochondrial dysfunction, which further caused apoptosis of oligodendrocyte progenitor cells, thereby compromising myelination [7]. Recent studies have reported that m^6^A methylation of mRNAs is involved in ER stress. The m^6^A methyltransferase WTAP increases ER stress by regulating m^6^A modification of ATF4 mRNA, thus promoting myocardial ischemia/reperfusion injury [31]. Another m^6^A methyltransferase, METTL14, accelerates the mRNA degradation of ER stress-related gene *CHOP*, thereby inhibiting the expression of proapoptotic target genes [32]. METTL14 can promote liver regeneration by mediating m^6^A modification of polypeptide-processing proteins in the ER [33]. By contrast, accumulation of unfolded or misfolded proteins in the ER lumen induces METTL14 expression by preventing its ubiquitination and degradation, which reveals a crosstalk between ER stress and m^6^A modification of mRNAs [32]. Based on previous studies and our results, we believe that m^6^A modification of the ER protein-processing pathway is closely related to bilirubin neurotoxicity and may form a basis for future research.

Functional analysis of differentially m^6^A-modified genes after UCB treatment of PC12 cells also showed the enrichment of the ubiquitin-mediated proteolysis in the KEGG pathway. The level of ubiquitinated proteins (Ub-prs) in the blood cells of patients with hyperbilirubinemia is significantly increased [34]. Bilirubin treatment induces the accumulation of Ub-prs in the brain of neonatal rats and inhibits the deubiquitination activity of the proteasome in a dose-dependent manner in vitro [34]. In our study, ubiquitin-mediated proteolysis was the top hypermethylation-affected pathway (Fig. 3c). These results suggested that m^6^A methylation might play a role in ER stress and that ubiquitin-mediated proteolysis was induced by bilirubin in neuronal cells.

In addition, in the pathways of ubiquitin-mediated proteolysis and protein processing in the ER, we selected the gene with the largest difference in m^6^A methylated peaks. Finally, we selected *Sect. 63* and *Ube2o* as the target genes for further study. Section 63 plays a key role in the transport of newly synthesized polypeptide precursors into the ER membrane by forming a dimeric complex with Sect. 62 [35]. The low expression of Sect. 63 leads to reduced myelination in both the central and peripheral nervous systems [36]. Ube2o is a member of the ubiquitin-conjugating enzyme family, which plays a vital role in the regulation of protein ubiquitination and cellular functions. Studies have reported that Ube2o has carcinogenic or tumor suppressive roles in human cancers [37–39]. However, whether Sect. 63 or Ube2o is involved in bilirubin neurotoxicity has not been reported and needs to be further elucidated.

Although our study presents some interesting findings and novel insights, it had some limitations. First, this study mainly focused on the m^6^A modification spectrum. We will further study and reveal the specific molecular mechanism involved in the changes and interactions of the enriched pathways in the m^6^A modification spectrum. Second, there may be differences between in vivo and in vitro m^6^A modifications induced by bilirubin in the CNS. In future studies, animal or human samples should be used to investigate changes in m^6^A modification levels in neonates with bilirubin-induced CNS injury.

## Conclusions

The present study confirmed that the profiles of mRNA m^6^A methylation were significantly altered in bilirubin-induced PC12 cell injury. These results suggested that m^6^A modification was involved in bilirubin-triggered CNS damage by regulating various pathways, such as ER stress, protease ubiquitination, and cell cycle. Our study provides new insights into the potential epigenetic mechanism of bilirubin neurotoxicity.

## Electronic supplementary material

Below is the link to the electronic supplementary material.

Supplementary Material 1

Supplementary Material 2

## Data Availability

The data and materials in the current study are available from the corresponding author upon reasonable request.
