# Supplementary figures and images for: Expression profiling of N6-methyladenosine-modified mRNA in PC12 cells in response to unconjugated bilirubin
Source: Mol Biol Rep. 2023 Jun 28;50(8):6703–15. doi: 10.1007/s11033-023-08576-1 (PMC10374823; doi:10.1007/s11033-023-08576-1)

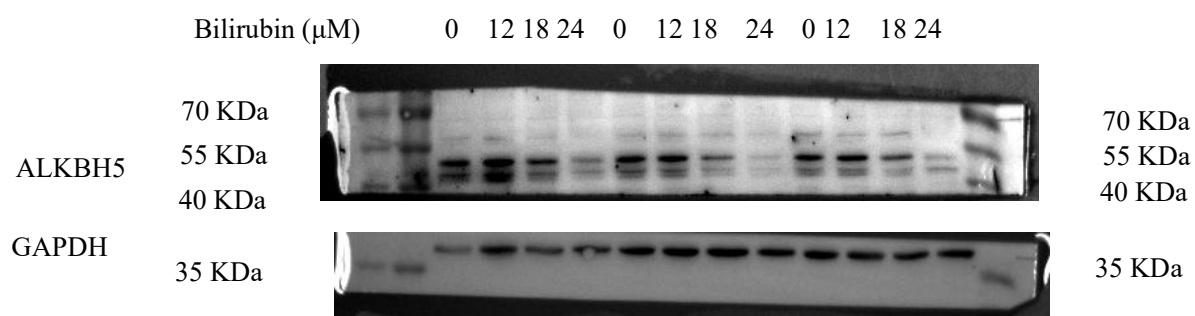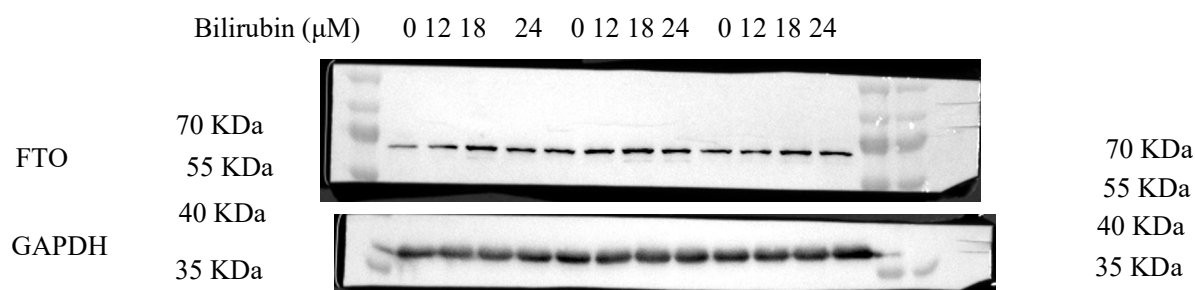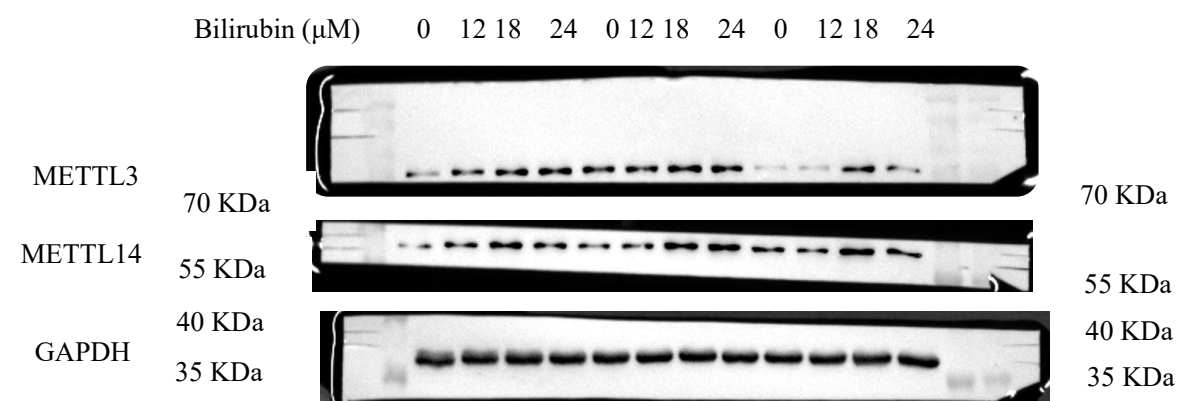

Supplement: Supplementary file 1 — Supplementary Material 1 [file 11033_2023_8576_MOESM1_ESM.pdf]
