# Supplementary material for: Expression profiling of N6-methyladenosine-modified mRNA in PC12 cells in response to unconjugated bilirubin
Source: Mol Biol Rep. 2023 Jun 28;50(8):6703–15. doi: 10.1007/s11033-023-08576-1 (PMC10374823; doi:10.1007/s11033-023-08576-1)
Supplement: Supplementary file 2 — Supplementary Material 2 [file 11033_2023_8576_MOESM2_ESM.pdf]

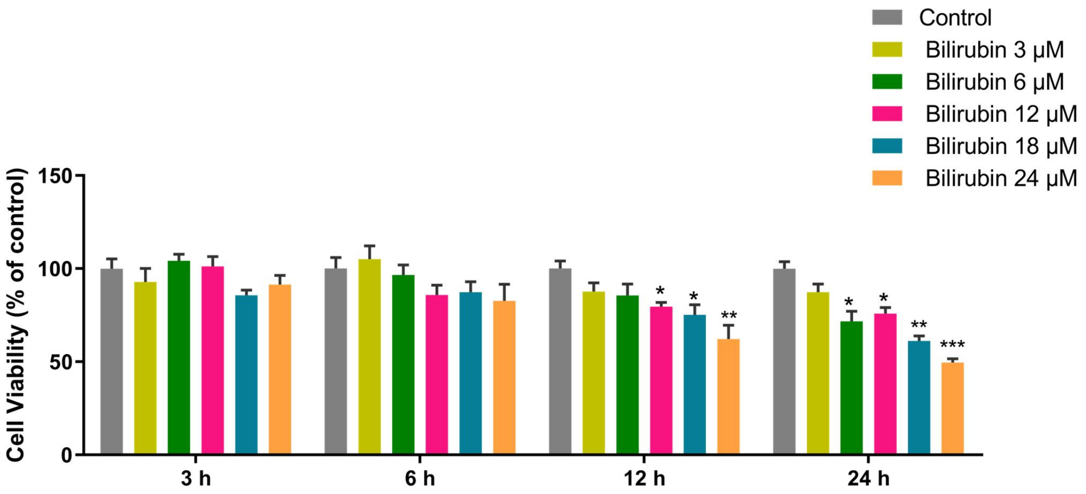

Fig. S1. The viability of PC12 cells treated with different concentrations of bilirubin for different time. PC12 cells were treated with different concentrations of bilirubin for different time. The data are presented as the mean ± SEM, n=6. *P < 0.05 and **P < 0.01 compared to the control group.
